# Performance Assessment of Ordered Porous Electrospun Honeycomb Fibers for the Removal of Atmospheric Polar Volatile Organic Compounds

**DOI:** 10.3390/nano8050350

**Published:** 2018-05-21

**Authors:** Yixin Wang, Hong Tao, Dengguang Yu, Changtang Chang

**Affiliations:** 1 School of Environment and Architecture, University of Shanghai for Science and Technology, Shanghai 200093, China; yixinwang@st.usst.edu.cn; 2 School of Materials Science and Engineering, University of Shanghai for Science and Technology, Shanghai 200093, China; ydg017@usst.edu.cn; 3 Department of Environmental Engineering, National I-Lan University, I-Lan 26047, Taiwan

**Keywords:** honeycomb fibers, ordered porous material, structural composite, VOC adsorption, recycling

## Abstract

This study explored a new facile method of preparing ordered porous electrospun honeycomb fibers to obtain the most promising composites for maximal adsorption of volatile organic compounds (VOCs). The self-assembly ordered porous material (OPM) and polyacrylonitrile (PAN) were formulated into a blend solution to prepare honeycomb fibers. SEM and TEM images showed that OPM was effectively bonded in PAN fibers because of the composite’s structure. Acetone was used as a model to assess the VOC adsorption performances of electrospun honeycomb fibers with different OPM contents. Experimental results revealed that the adsorption capacity of honeycomb fibers increased with the increase of loaded OPM within the PAN fibers. The highest adsorption capacity was 58.2 μg g^−1^ by the fibers containing with 60% OPM in weight. After several recycling times, the adsorption capacities of the reused honeycomb fibers were almost the same with the fresh fibers. This finding indicated that the electrospun honeycomb fibers have potential application in removing VOCs in the workplace, and promote the performance of masks for odor removal.

## 1. Introduction

Volatile organic compounds (VOCs) are emitted mainly by industrial enterprises and seriously pollute the environment [1]. Thus, an economic and effective way to control VOCs is important to reduce human-health risks and environmental damage [2,3]. Adsorption is a widely used method of rapidly and effectively removing VOCs because other VOC-removal technologies (e.g., absorption, catalytic reactions, photoreactions, and biological filtration) are difficult to apply because of their high cost or low efficiency [4,5,6,7,8]. VOC removal through traditional adsorbents such as activated carbon, graphene oxide, and metal organic frameworks [3,9,10,11] is difficult for industrial practice presently because recycling or reusing adsorbents is not easy. Electrospun fibers, which are often assembled in the format of non-woven mats, hold great promise for applications as efficient adsorbents and also other functional nanomaterials because of their unique properties such as huge surface area, larger porosity and 3-D web structure [12,13,14,15].

Atmospheric VOCs can be effectively removed by electrospun fibers, which have adsorption abilities reportedly similar to that of activated carbon [16,17]. In addition, different non-spun solid particles can be added to effectively increase the adsorption capacity of electrospun fibers [16,17]. In recent years, electrospun polyacrylonitrile (PAN) fibers have drawn a great deal of attention due to their good thermal stability, fine precursors of carbon nanofibers and excellent filament-forming properties [18,19]. In the literature, electrospun fibers were reported to be used successfully for removing nonpolar or weak polar VOCs, such as toluene, benzene and ethanol [20,21,22]. Thus, it is anticipated that electrospun PAN fibers can be explored to remove acetone from the atmosphere.

The use of fibers in combination with porous materials can enhance the performance of VOCs adsorption and reuse properties of resultant composites. In the present study, the binding product of self-assembled ordered porous material (OPM) and electrospun fiber is proposed as honeycomb fiber according to its structure for the first time. The surface chemical properties, functional groups and fiber structures of OPM are satisfied to meet the adsorption requirements [23]. The binding properties of different OPM contents and electrospun PAN fibers were evaluated to obtain optimal honeycomb fibers with the highest adsorption capacity. The adsorption mechanism is investigated by exploring the key reaction of acetone removal. The recycling performance of renewable honeycomb fibers was also evaluated to effectively develop environmentally friendly functional materials.

## 2. Materials and Methods

### 2.1. Materials

Polyacrylonitrile (PAN, *M*_w_ = 150,000) was obtained from Sigma Company in USA and *N*,*N*-dimethylformamide (DMF) was chosen as the solvent. Tetraethylorthosilicate (TEOS, 98%) and cetyltrimethylammonium bromide (CTMABr, 99%) were obtained from Aldrich Company in USA. Isopropanol, NaOH and aqueous ammonia (28%) were purchased from Shiyaku Company in Japan.

### 2.2. Preparation of OPM

Sol-gel method was used for OPM self-assembly synthesis. Cetyl trimethyl ammonium bromide (CTMABr) of 2.5 g was homogenized in NaOH of 10 mL and deionized of 125 mL water before adding NH_4_OH (28% by weight) of 10 mL. The mixture was stirred at room temperature under magnetic stirring for 30 min until CTMABr was completely dissolved. Then, 5.2 mL TEOS and 10.4 mL Propan-2-ol (isopropanol) were added dropwise to the aqueous CTMABr solution and stirred at room temperature for 8 h. The stirred sample solution was collected by suction filtration. Then, the white precipitate was collected and placed in an oven dried at 120 °C overnight to yield the as-synthesized OPM product. The dried solid product was placed in a high-temperature furnace and calcined at 550 °C for 6 h to remove the organic template. The obtained OPM was naturally cooled to room temperature and placed in a dry oven for preservation.

### 2.3. Synthesis of Electrospun Honeycomb Fibers Composites

The prepared OPM was ground to a powder (using a diameter of 74 μm sieve) and placed in a drying oven at 80 °C for at least 4 h. The different contents (20, 40, 60 and 80 wt % relative to PAN) of OPM powder were dispersed in DMF and sonicated for 2 h to produce a homogeneous solution. Then, PAN was added to solution and followed by magnetic stirring for another 5 h. The OPM-PAN composite nanofibers were prepared and named as 20% M-P, 40% M-P, 60% M-P and 80% M-P, respectively. Electrospinning parameters included 13 kV applied voltage, 18 cm collection distance and 0.5 mL h^−1^ fluid flow rate. After electrospinning, the peeled-off fiber mats were dried at 50 °C for 18 h in a vacuum. Given the special structure of the composite’s fibers, they can be called honeycomb fibers.

### 2.4. Morphology

The morphological structure of the honeycomb fiber was characterized with using a field emission scanning electron microscope (FE-SEM, Hitachi, Tokyo, Japan). Furthermore, the elemental composition of the sample was analyzed with using an energy dispersive X-ray spectrometer connected to a SEM (EDS, IE300X, Oxford Instruments, Oxford, UK). Transmission electron microscopy images of fibers (TEM, Hitachi, Japan) were recorded at an accelerating voltage of 100 kV. The TEM samples were collected by using a copper mesh with a carbon layer on the collector for several minutes during the electrospinning processes.

### 2.5. Physical Forms of the Components and Their Interactions

The X-ray diffraction pattern (XRD) of the honeycomb fiber was obtained by using a Rigaku D/MAX-B diffractometer equipped with Cu Kα radiation at λ = 0.154 nm. The diffract spectra of the fibers were recorded within a 2θ range from 0° to 60° with each point recorded in a step of 0.5° and a count time of 60 s. The prepared fibers were subjected to attenuated total reflection Fourier transform infrared (ATR FTIR) analysis with a resolution of 2 cm^−1^ with using a Nicolet-Nexus 670 FTIR spectrometer (Nicolet Instrument Corporation, Madison, WI, USA) within the range of 600–4000 cm^−1^.

### 2.6. Hydrophobic Property

The hydrophobicity of the honeycomb fiber mats were determined using a droplet analysis instrument (DSA100, Krüss GmbH, Hamburg, Germany) by measuring the surface water contact angle (WCA). For each sample, a droplet of 3 μL double-distilled water was placed on their surface, which was repeated six times.

### 2.7. VOC Adsorption Performance Assessment

**Figure 1:**
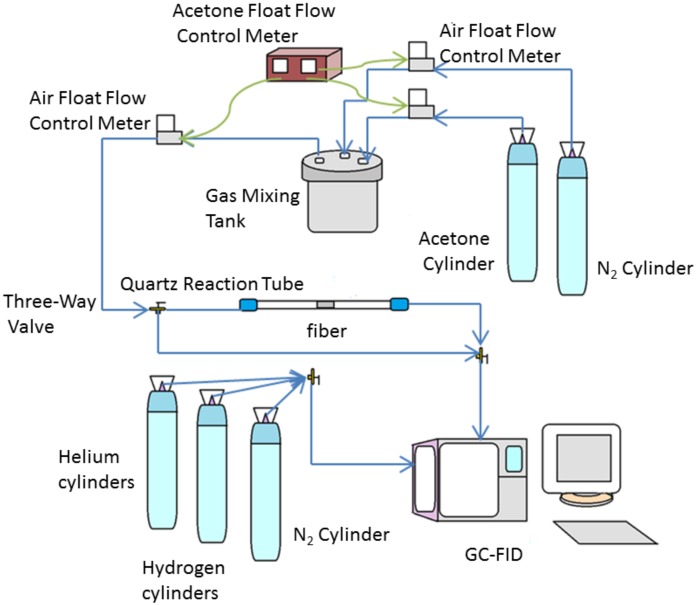
Diagram of performance assessment system.

Acetone is a representative of polar molecules and is used to evaluate the VOC-adsorption capacity of different electrospun honeycomb fibers. The dynamic adsorption and desorption processes were examined according to the diagram shown in Figure 1. The OPM-PAN composite fibers, 0.88 g∙cm^−3^, were placed in a quartz reaction tube, whose ends were both connected with a pipeline to form a continuous reactor. The initial acetone concentration at the reactor inlet was controlled as 100 ppm and the flow rate was set at 1 mL min^−1^. The reaction outlet was connected to a gas chromatograph with flame ionization detector (GC-FID) to measure acetone concentration. The VOCs adsorption breakthrough curve of each fiber was obtained at room temperature, and the adsorption capacity of the honeycomb fibers were calculated. In the performance analysis of honeycomb fibers, N_2_ was used to purge the fibers to remove the acetone molecules adsorbed in the fibers under 105 °C. Residual acetone in the honeycomb fibers was determined by GC-FID after desorption. All the experiments were repeated three times.

### 2.8. Adsorption Isotherms

#### 2.8.1. Langmuir Isotherm

The Langmuir model, as shown in Equation (1), was used to correlate the experimental equilibrium data considering monolayer adsorption on a uniform surface containing a finite number of uniform adsorption sites within the polymer, which means that no further adsorption occurs at this site [24]. The maximum adsorption of the surface can be obtained when the surface reaches saturation. In addition, it is assumed that the polymer has a uniform structure in which the adsorption sites are equivalent in energy [25].

(1) $$q_{e}=\frac{q_{m}K_{d}C_{0}}{1+K_{d}C_{0}}$$

where, *C*_0_ (ppm) and *q_e_* (μg g^−1^) is the initial concentration and adsorption capacity, respectively; *q_m_* and *K_d_* are the Langmuir isotherm constants. It can be used to predict the affinity between VOCs and honeycomb fibers, and the Langmuir constant is used as the separation factor or the dimensionless equilibrium parameter, which is expressed as follows in Equation (2).

(2) $$R_{L}=\frac{1}{1+K_{d}C_{0}}$$

where, *R_L_* is the Langmuir constant and indicates the adsorption property. The adsorption process is irreversible, favorable, linear and unfavorable reaction when *R_L_* is equal to 0, between 0 and 1, equal to 1 and larger than 1.

#### 2.8.2. Freundlich Isotherm

The Freundlich model shows the type of adsorption on the heterogeneous surface that interacts with adsorbed molecules. The adsorption energy decreases exponentially according to the adsorption center of the polymer and the completion of the reversible adsorption and is not limited to the formation of a monolayer. The Freundlich isotherm is expressed as follows in Equation (3) [26].

(3) $$\ln q_{e}=\ln K_{f}+n\ln C_{0}$$

where, *K_f_* is the Freundlich constant, and *n* is heterogeneity.

#### 2.8.3. Temkin Isotherm

According to the characteristics of the Temkin isotherm, it is assumed that the binding energy on the honeycomb fiber surface is uniformly distributed. This model also assumes that the adsorption heat associated with their interaction decreases linearly as increasing acetone capacity. In addition, the specific recognition sites are randomly distributed inside the inner cavities of honeycomb fibers. The Temkin isotherms are listed as follows in Equation (4).

(4) $$Q_{eq}=B_{T}\ln\left(A_{T}\right)+B_{T}\ln\left(C_{eq}\right)$$

where, *A_T_* is the Temkin isotherm constant (J g^−1^) that represents the maximum binding energy and *B_T_* is the Temkin isotherm constant (J mol^−1^) and related to the heat of adsorption.

## 3. Results and Discussion

### 3.1. Electrospinning

**Figure 2:**
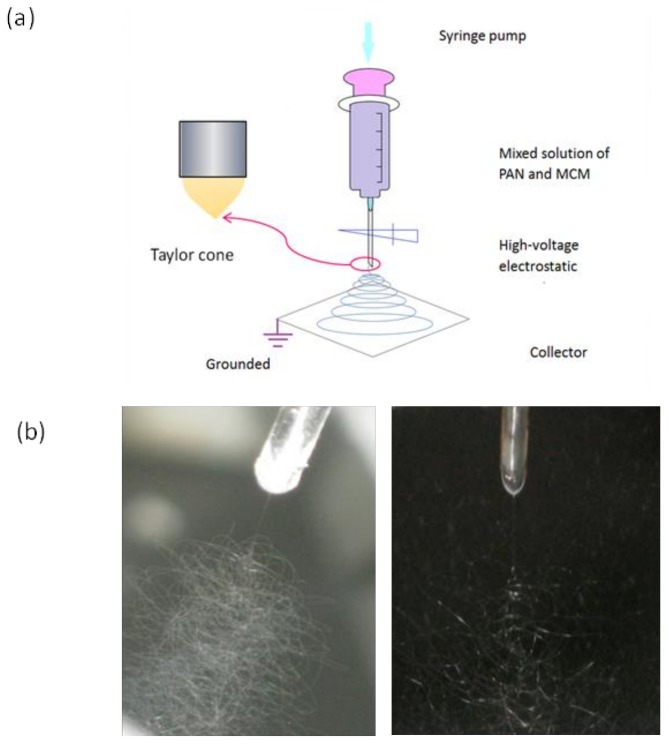
(**a**) A diagram of the electrospinning system; (**b**) Two typical digital images about the working processes during electrospinning.

Electrospinning is generally considered to be a cheap, simple and straightforward process for preparing polymeric nanofiber and fiber-based nanocomposites, which hold a large surface area and high porosity in nature [27,28,29]. A diagram of the electrospinning process and the visual digital photos captured during the working processes are shown in Figure 2. The electrospinning system includes a syringe pump for driving the PAN and OPM mixture solution in the syringe. The high-voltage power supply is connected with the metal spinneret via an alligator clip. The fibers were collected on a grounded plate collector with an aluminum foil cover.

### 3.2. Characterization

#### 3.2.1. Surface Area and Pore Size Distribution

The specific surface areas and pore sizes of the samples from 20% M-P to 60% M-P increased with the increase of OPM contents within the electrospun nanofibers, which are included in Table 1. However, the pore volume of 60% M-P was almost double that of 80% M-P. This abnormal phenomenon should have a close relationship with the agglomeration of OPM and the entrapment of OPM by PAN.

#### 3.2.2. Morphology

**Figure 3:**
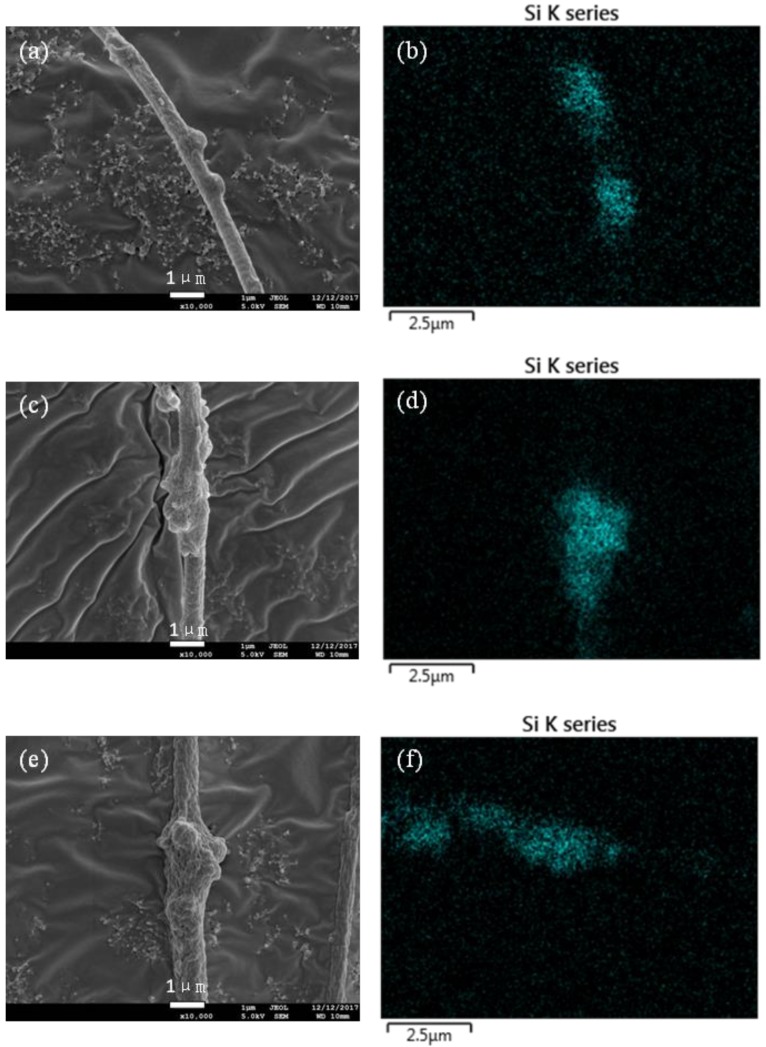

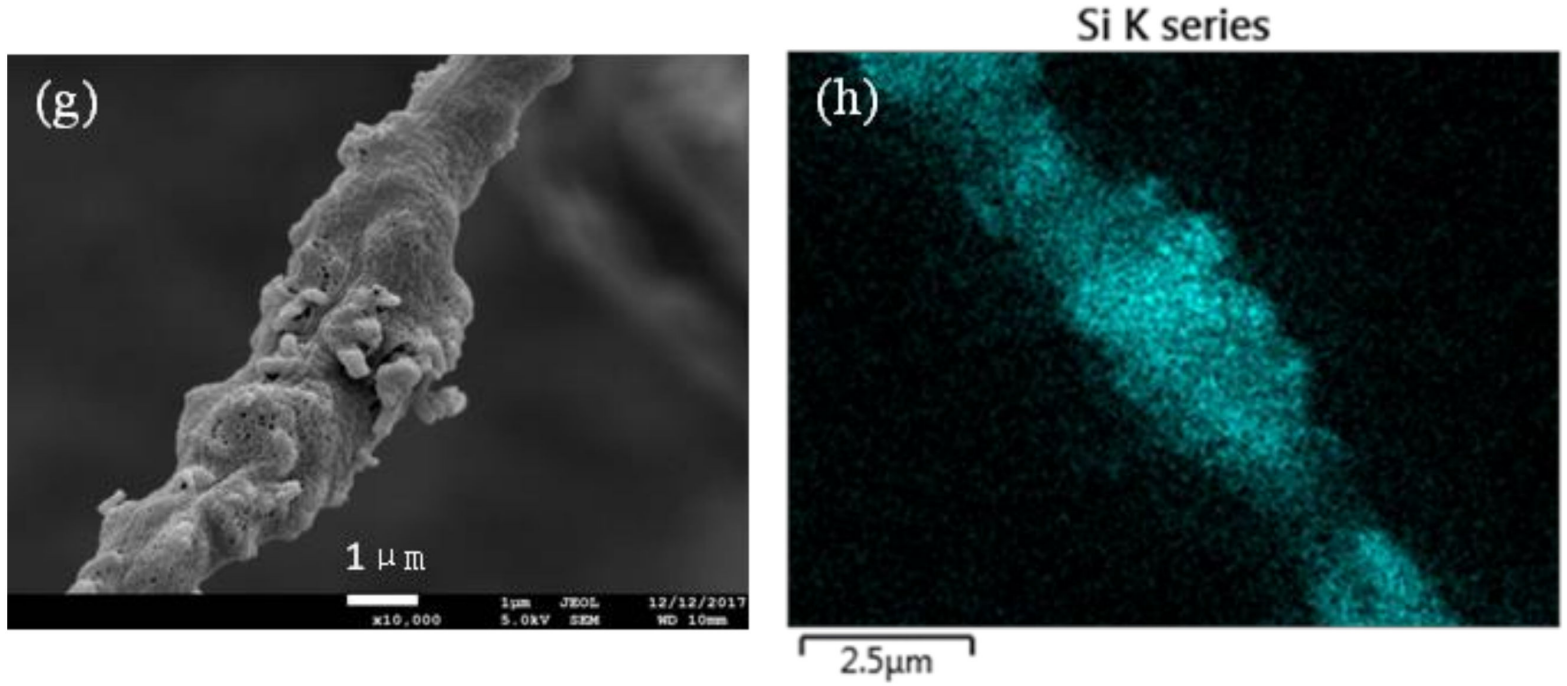
Morphological images of single honeycomb fibers: (**a**) 20% M-P; (**b**) 20% M-P-EDS; (**c**) 40% M-P; (**d**) 40% M-P-EDS; (**e**) 60% M-P; (**f**) 60% M-P-EDS; (**g**) 80% M-P; (**h**) 80% M-P-EDS.

The prepared honeycomb nanofibers are deposited randomly on the collector to form a flexible and continuous non-woven mat. FE-SEM images of these honeycomb fibers with different OPM contents are shown in Figure 3. The homogeneous distributions of OPM within the PAN matrix ensured a porous surface structure of the OPM-PAN composite nanofibers, i.e., honeycomb surface morphologies. The diameter of fibers increases with increased OPM content, as shown in Table 1. Based on the fiber morphology observed with SEM, the addition of OPM also contributed to the high specific surface area of the composite honeycomb fiber mats. OPM content could not be too high. This is because of the requirement of generating uniform nanofibers, and because a high content of 80% OPM frequently clogged the nozzle of spinneret. The fibers were relatively smooth, and the frequency of nodes on the fiber was relatively low when the OPM content was smaller than 60%. Besides the increase of fiber diameter with the increase of OPM, it is also obvious that the fibers’ smoothness decreased gradually due to more OPM on the fibers’ surfaces, which was indicated in Figure 3a–h. The analysis of Si by FE-SEM-EDS similarly suggested that the element Si was effectively loaded on the fibers. Due to the easy agglomeration of non-spinning OPM, the amount of fiber nodes increased with the elevations of OPM.

**Figure 4:**
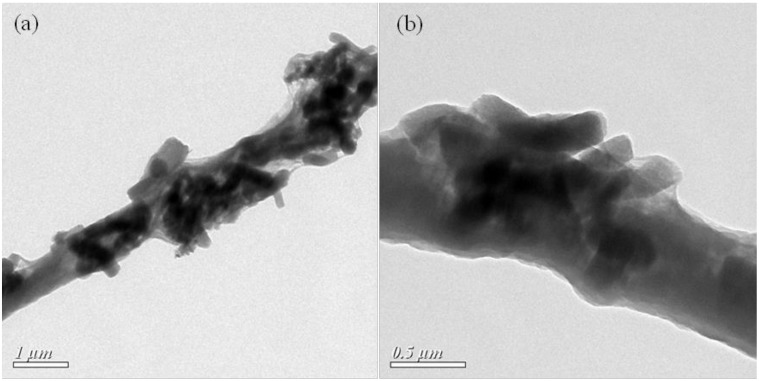
TEM images of 60% M-P: (**a**) fiber and (**b**) node.

The TEM images of 60% M-P are shown in Figure 4a,b. From Figure 4a, it is clear that some parts of the dispersed OPM were wrapped inside the PAN matrices, and the other parts were successfully exposed on the surface of composite nanofibers. Figure 4b is an image about a node, within which OPM penetrated the PAN fiber and roughened the fiber’s surface.

#### 3.2.3. Physical Forms

**Figure 5:**
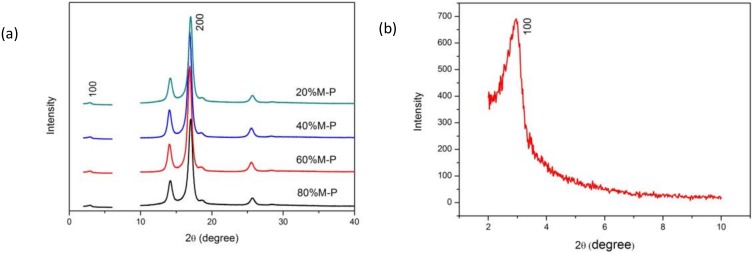
XRD patterns of (**a**) honeycomb composite fibers and (**b**) OPM.

The XDR patterns of honeycomb fibers with different contents of OPM are compared in Figure 5a. The diffraction peaks at 2θ = 17° are suggested to be the reflections of the characteristics of the (200) crystal plane of the originally existing C–N groups in PAN. The low-angle peak of mesoporous OPM is shown in Figure 5b. The XRD patterns of composite nanofibers show both the diffraction peaks (1 0 0) at 2θ = 3.0° (attributed to the characteristics of the hexagonal mesoporous structure of spherical particles [30]) and also the diffraction peaks at 2θ = 17°, indicating a co-present state of OPM and PAN within the electrospun nanofibers.

#### 3.2.4. Functional Groups

**Figure 6:**
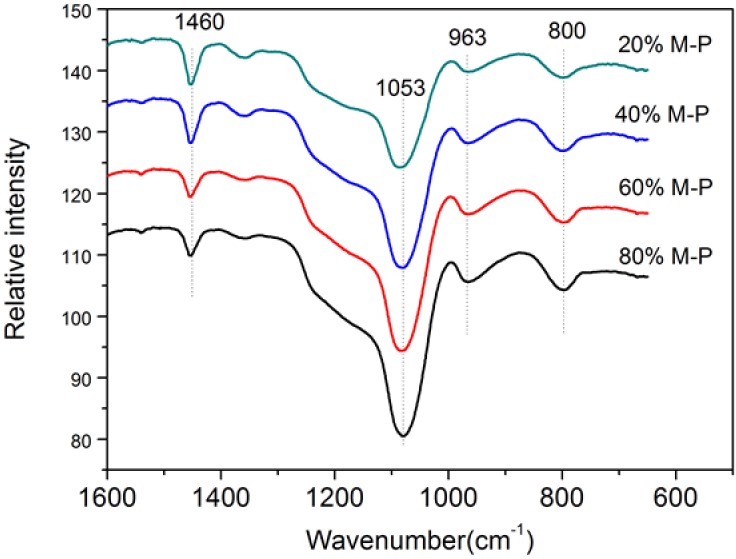
FT-IR spectra of the honeycomb fibers.

FT-IR spectroscopy was used to analyze the function group of fiber mats. Shown in Figure 6, the wavenumber of Si–O stretching vibration bands and Si–O asymmetric stretching vibration bands is located at 800 cm^−1^ and 1053 cm^−1^, respectively, which has also reported in literature [31]. Si–O is a characteristic functional group of OPM. These peaks indicates the presence of OPM in the composite fibers. The corresponding Si–O peak increases with increased content of OPM, suggesting that OPM was effectively attached onto the fiber surface. The ≡Si–O–H telescopic vibration band is located at 963 cm^−1^, verifying the presence of silanol groups on the fibers’ surfaces [32]. The rotating vibration bands of –CH groups are located at 1460 cm^−1^, reflecting the presence of PAN in the composite fibers [33].

#### 3.2.5. Hydrophobic Property

**Figure 7:**
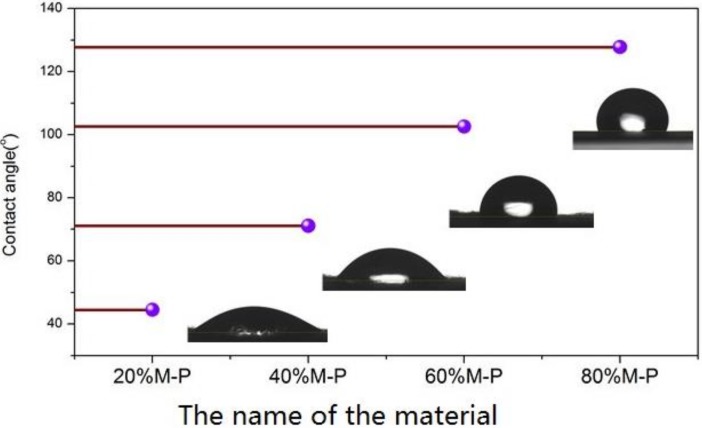
Contact angles of honeycomb fibers with different OPM contents.

To investigate the hydrophobic property of the fiber surface, the contact angles of honeycomb fiber mats were measured and the results are shown in Figure 7. Higher contact angle means better hydrophobic property, which is important for the honeycomb composite fiber for avoiding the negative influence of moisture on polar VOC adsorption. From changes of contact angle from 44.5° to 127.8°, the results demonstrated that the hydrophobic performances were improved as the increase of OPM contents within the composite nanofibers, which should correspondingly improve the performance of honeycomb fibers for acetone removal.

### 3.3. Adsorption Performance of Honeycomb Fibers

**Figure 8:**
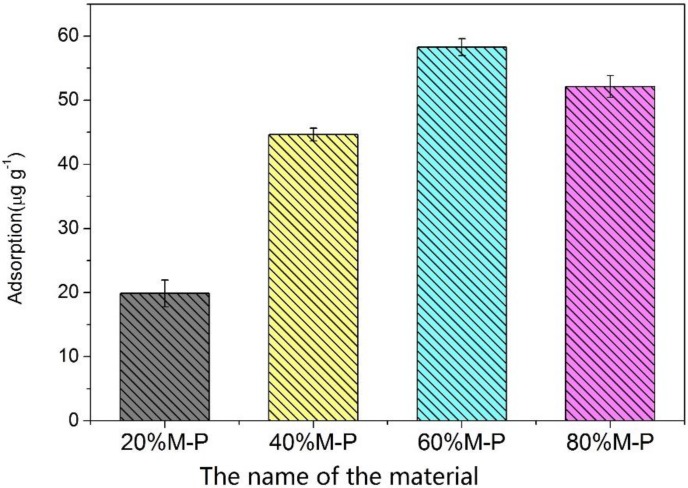
Adsorption capacity of honeycomb fibers with different OPM contents (*n* = 3).

The adsorption capacity of honeycomb fibers with different OPM contents for acetone removal is shown in Figure 8. The acetone adsorption efficiency was evaluated at a flow rate of 1 mL min^−1^ and acetone concentration of 100 ppm to find the optimal OPM content in the composite nanofibers. The adsorption capacity of acetone was evaluated to determine the best OPM content. It is obvious that the adsorption capacity of honeycomb fibers for acetone removal increases with increasing OPM content. In Figure 8, the best adsorption capacity of 58.2 μg g^−1^ is to use 60% M-P fiber for acetone removal, which is significantly higher than that of 40% M-P and 20% M-P. These results can be anticipated because OPM has a mesopore structure and is suitable for acetone adsorption [34,35]. T Thus, the more OPM was loaded, the better adsorption performance the composite fibers had. However, this trend was broken by the 80% M-P fiber, whose adsorption capacity is 52.1 μg g^−1^, less than that of 60% M-P fiber (58.2 μg g^−1^). The reason should be that the pore volume of 60% M-P was almost twice than that of 80% M-P, where the agglomeration of OPM was severe.

### 3.4. Effect of Adsorption Concentration

**Figure 9:**
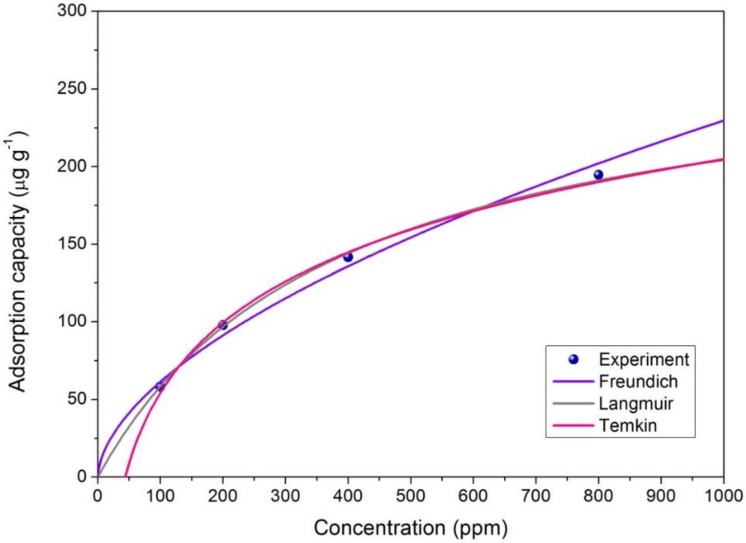
Adsorption capacity estimation for acetone adsorption (*n* = 3).

In Figure 9, the Langmuir, Freundlich and Temkin isotherm models of acetone adsorption with 60% M-P fibers are fitted. The Langmuir adsorption isotherm model (*R*^2^ = 0.9988) is more suitable for adsorption capacity prediction than the Freundlich adsorption isotherm model (*R*^2^ = 0.9880) and Temkin adsorption isotherm model (*R*^2^ = 0.9925) at acetone concentrations of 100, 200, 400, 800 ppm. The calculated parameters according to these models are included in Table 2. The evaluated R_L_ is about 0.27, which suggests that the acetone removal process belonged to favorable adsorption (0 < *R_L_* < 1). The results also confirmed that the adsorption of acetone by composite fibers was monolayer and physical in nature.

### 3.5. Reusability

**Figure 10:**
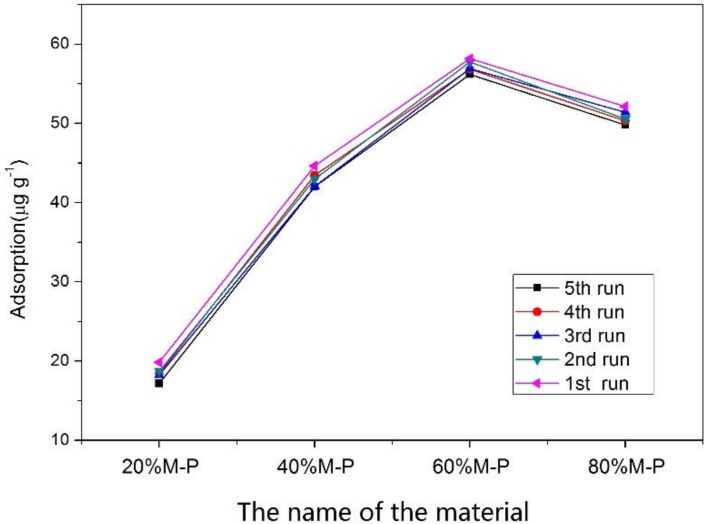
The influences of recycling times on the acetone adsorption by honeycomb fibers (*n* = 3).

Most of the adsorbent material in the form of particles and powder can limit their re-use and commercial value. In this study, OPM was added into PAN fiber by electrospinning, and the honeycomb fibers were prepared to enhance the VOC adsorption performance through increasing the specific surface area and pore volume of the fiber at the same time. Furthermore, acetone could be desorbed from OPM at the lower temperature [30]. The four kinds of honeycomb fibers were used to repeat the VOC adsorption/desorption experiments for five recycling times, and the results are shown in Figure 10. The adsorption data revealed that the results of honeycomb fibers with different OPM contents were similar with each run during the adsorption and desorption processes. Furthermore, 60% M-P honeycomb fibers always had the best adsorption capacity of around 58.3 μg g^−1^ for acetone removal. After 5 repetitions, the differences about the adsorption performance between the first and the fifth run was less than 5%. These results showed better performances about reusability than those fly-ash-loaded fibers and activated carbon reported in literature [16,17].

### 3.6. Temperature Effect on Adsorption Performance

**Figure 11:**
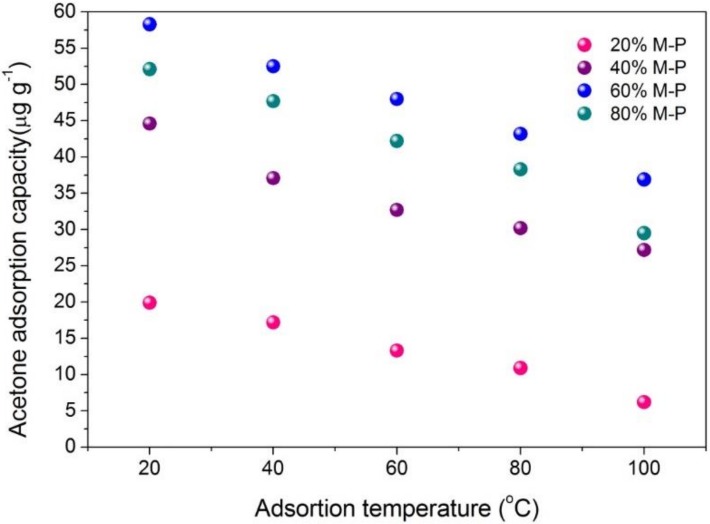
The influence of temperature on equilibrium acetone adsorption capacity of honeycomb fibers (*n* = 3).

**Figure 12:**
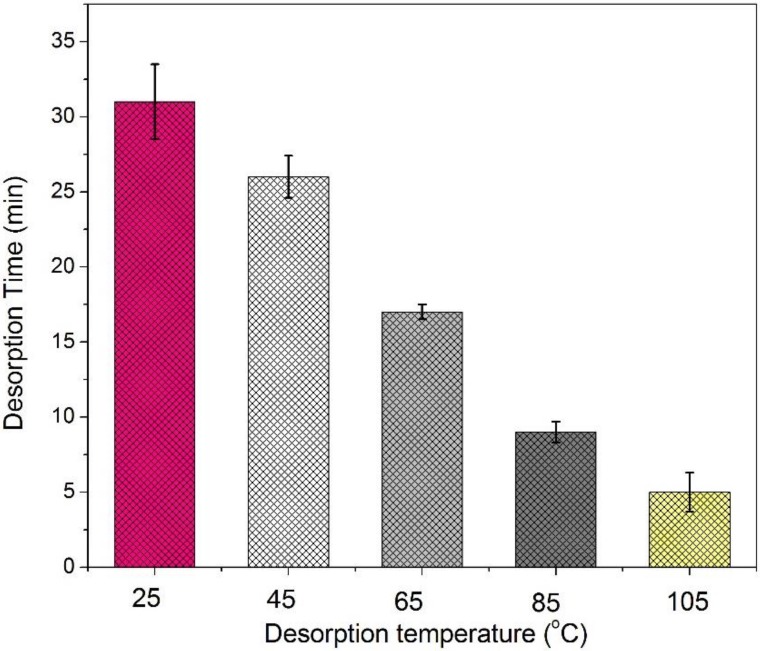
Desorption time of 60% M-P at different temperatures (*n* = 3).

The effect of temperature on the acetone adsorption capacity of the honeycomb fibers were assessed in the range of 20–100 °C and the results are shown in Figure 11. The adsorption capacity was relatively large at the lower temperature and gradually decreased with increasing temperature. The relationships between adsorption capacity and temperature for the four honeycomb fibers are also similar. At an adsorption temperature of 20 °C, the 60% M-P adsorption capacity was approximately 58.3 μg g^−1^ and decreased to an approximate value of 51.9 μg g^−1^ at 40 °C. While investigating the influence of temperature on the adsorption of acetone, desorption experiments were also conducted. As the temperature rose, the rate of which acetone resolving increased and the time taken for the desorption process gradually shortened (Figure 12).

### 3.7. Development of Adsorption Mechanism

**Figure 13:**
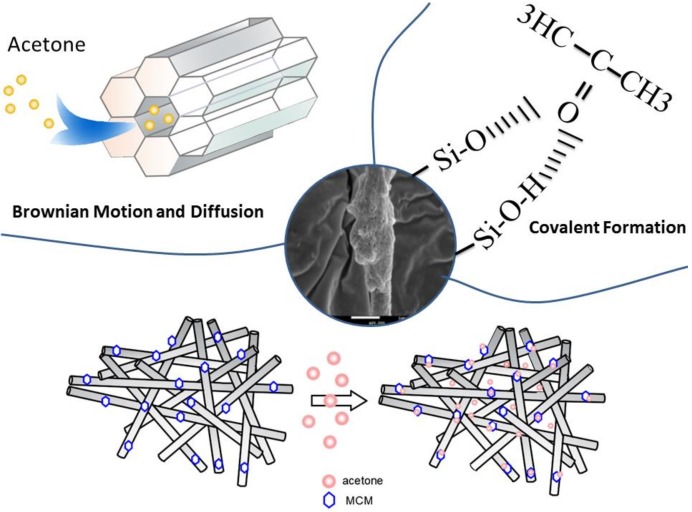
A diagram about the honeycomb fiber adsorption process of acetone.

Based on the experimental data and characterization studies, honeycomb fiber can be used for acetone adsorption effectively since the OPM penetrates the fiber and is exposed to the outside of the fiber with a large area for effective acetone adsorption. This is clearly demonstrated by the SEM image of the single fiber. Furthermore, the Si–O functional group on the honeycomb fiber can be effectively combined with C=O functional group of acetone, which increase the adsorption capacity of acetone, as shown in Figure 13. In addition, the superposition of fibrous layers also creates higher porosity which helps to capture acetone efficiently.

It can be speculated that the removal mechanism consists of three steps. In First step, acetone is adsorbed on the surface of honeycomb fiber due to Brownian motion. In second step, the partial acetone is diffused into inner pores of honeycomb fiber due to core adsorption sites provided by porous materials of honeycomb fiber. In third step, acetone is adsorbed through the intermolecular interactions between carboxyl and Si–O as verified by the EDS results (Figure 3 and Figure 6).

## 4. Conclusions

In summary, new types of honeycomb fiber were prepared successfully and were explored for effective polar VOC removal. The size and structure estimated by SEM and TEM demonstrated the uniform dispersion of OPM in honeycomb fibers. The description of its physico-chemical properties (BET, FT-IR, XRD and contact angle) indicated that the best-performance honeycomb fiber is 60% M-P with the largest pore volume. In addition, the composite fibers had excellent recycling performances. These honeycomb fibers may find potential applications as absorbents in plants to remove VOCs or as commercial masks.

## Figures and Tables

**Table 1:** Properties of OPM-PAN fibers with different contents of OPM.

Sample	S_BET_^a^	Pore Volume	Pore Size	Fiber Diameter (nm)	
	(m^2^g^−1^)	(m^2^g)	(nm)	Range	Mean
20% M-P	62	0.02	2.12	52–90	65
40% M-P	113	0.03	2.16	68–184	103
60% M-P	121	0.05	2.19	71–218	119
80% M-P	219	0.03	3.66	113–306	199

S_BET_^a^: specific surface area calculated by Brunauer–Emmett–Teller (BET) method.

**Table 2:** Langmuir, Freundlich and Temkin constants for acetone adsorption with 60% M-P.

Adsorption	Langmuir Isotherm			Freundlich Isotherm			Temkin Isotherm		
	*K_d_*	*q_m_*	*R* ^2^	*K_f_*	*n*	*R* ^2^	*B_T_*	*A_T_*	*R* ^2^
60% M-P	0.0036	277.78	0.9988	4.39	1.75	0.9880	65.36	0.023	0.9925
